# Ultra-Rare Mutation in Long-Range Enhancer Predisposes to Thyroid Carcinoma with High Penetrance

**DOI:** 10.1371/journal.pone.0061920

**Published:** 2013-05-14

**Authors:** Huiling He, Wei Li, Dayong Wu, Rebecca Nagy, Sandya Liyanarachchi, Keiko Akagi, Jaroslaw Jendrzejewski, Hong Jiao, Kevin Hoag, Bernard Wen, Mukund Srinivas, Gavisha Waidyaratne, Rui Wang, Anna Wojcicka, Ilene R. Lattimer, Elzbieta Stachlewska, Malgorzata Czetwertynska, Joanna Dlugosinska, Wojciech Gierlikowski, Rafal Ploski, Marek Krawczyk, Krystian Jazdzewski, Juha Kere, David E. Symer, Victor Jin, Qianben Wang, Albert de la Chapelle

**Affiliations:** 1 Human Cancer Genetics Program and Department of Molecular Virology, Immunology and Medical Genetics, Ohio State University Wexner Medical Center and Comprehensive Cancer Center, the Ohio State University, Columbus, Ohio, United States of America; 2 Department of Molecular and Cellular Biochemistry, Ohio State University Wexner Medical Center and Comprehensive Cancer Center, the Ohio State University, Columbus, Ohio, United States of America; 3 Department of Internal Medicine, Ohio State University Wexner Medical Center and Comprehensive Cancer Center, the Ohio State University, Columbus, Ohio, United States of America; 4 Department of Biomedical Informatics, Ohio State University Wexner Medical Center and Comprehensive Cancer Center, the Ohio State University, Columbus, Ohio, United States of America; 5 Department of Biosciences and Nutrition, Clinical Research Centre, and Science for Life Laboratory, Karolinska Institutet, Stockholm, Sweden; 6 Department of Endocrine Surgery, Maria Sklodowska-Curie Memorial Cancer Center and Institute of Oncology, Warsaw, Poland; 7 Department of Nuclear Medicine and Endocrine Oncology, Maria Sklodowska-Curie Memorial Cancer Center and Institute of Oncology, Warsaw, Poland; 8 Department of Biochemistry and Molecular Biology, Medical Centre of Postgraduate Education, Warsaw, Poland; 9 Genomic Medicine, Department of General, Transplant, and Liver Surgery, Medical University of Warsaw, Warsaw, Poland; 10 Department of Medical Genetics, Medical University of Warsaw, Warsaw, Poland; 11 Folkhälsan Institute of Genetics, Helsinki, and Research Program's Unit, University of Helsinki, Helsinki, Finland; MOE Key Laboratory of Environment and Health, School of Public Health, Tongji Medical College, Huazhong University of Science and Technology, China

## Abstract

Thyroid cancer shows high heritability but causative genes remain largely unknown. According to a common hypothesis the genetic predisposition to thyroid cancer is highly heterogeneous; being in part due to many different rare alleles. Here we used linkage analysis and targeted deep sequencing to detect a novel single-nucleotide mutation in chromosome 4q32 (4q32A>C) in a large pedigree displaying non-medullary thyroid carcinoma (NMTC). This mutation is generally ultra-rare; it was not found in 38 NMTC families, in 2676 sporadic NMTC cases or 2470 controls. The mutation is located in a long-range enhancer element whose ability to bind the transcription factors POU2F and YY1 is significantly impaired, with decreased activity in the presence of the C- allele compared with the wild type A-allele. An enhancer RNA (eRNA) is transcribed in thyroid tissue from this region and is greatly downregulated in NMTC tumors. We suggest that this is an example of an ultra-rare mutation predisposing to thyroid cancer with high penetrance.

## Introduction

Thyroid cancer represents approximately 1% of newly diagnosed cancers and is the most common endocrine malignancy. There are four main varieties of thyroid cancer, papillary (PTC), follicular (FTC), medullary (MTC), and anaplastic (ATC). The majority of all thyroid tumors are non-medullary thyroid carcinoma (NMTC); either PTC (80–85%) or FTC (10–15%). Contrary to many other cancers the incidence of NMTC is increasing in recent decades [1], [2]. While the etiology of NMTC is not well characterized, it is clearly influenced by both genetic and environmental factors. Among the latter, ionizing radiation, especially exposure to fallout of radioactive iodine isotopes in childhood strongly predisposes to PTC [3]. On the other hand, genetic predisposition plays a major role as evidenced by case control studies [4], [5]. NMTC is mostly sporadic; however increasingly over the past 20 years, the occurrence of NMTC running in families has been observed [6]. Large population-based studies investigating the familial aggregation of the disease indicate a significantly increased risk of NMTC among first degree relatives [7]–[9]. It has been estimated that 5 to 10% of all NMTC are “familial” [4], [10]. The familial form of NMTC has been recognized as a distinct clinical entity with a more severe phenotype than its sporadic counterpart [11], [12]. Usually the familial NMTC pedigrees are small with 3 or fewer affected individuals; autosomal dominant inheritance with reduced penetrance is usually suggested in these families. In the past these findings provided the rationale for linkage studies in NMTC families, which identified at least 7 different genomic regions on chromosomes 1q21 [13], 2q21 [14], 6q22 [15], 8p23 [16], 8q24 [17], 14q31 [18], and 19p32 [19] showing linkage peaks presumably harboring predisposing genes. In most cases no predisposing gene mutation has been described. Indeed, the genetic factors influencing susceptibility to NMTC (high or medium penentrance) remain largely unknown. In contrast, genome-wide association studies have disclosed low-penetrance loci predisposing to thyroid cancer [20], [21]. We begin to understand the molecular basis of this type of predisposition; at least in one case a lincRNA is involved [22].

According to a common hypothesis the genetic susceptibility of complex disorders such as cancer may be highly heterogeneous in part due to many different rare alleles. Such alleles have not been described in thyroid cancer. We present here an example of such an allele. We identified a large US mid-western family with 13 individuals diagnosed with NMTC in three generations. We conducted genome-wide linkage analyses and found strong evidence of linkage at chromosome 4q32. We show that a long-range enhancer element is present within the linkage peak. A single nucleotide mutation (4q32 A>C) affects the binding of transcription factors POU2F1 (also called OCT1) and YY1 to a DNA motif in the enhancer and significantly alters the luciferase reporter activity. Moreover, an enhancer RNA (eRNA) was detected in normal thyroid tissue in the mutation region and the eRNA expression level was strongly reduced in NMTC tumors. Taken together, evidence from linkage, chromatin signature, luciferase reporter assays, and gene expression analysis suggested a long-range enhancer at 4q32 as a candidate genetic factor for the high penetrance PTC predisposition in the family. The mutation is ultra-rare not having been found in databases, 38 other familial NMTC kindreds, sporadic thyroid cancer patients, or controls.

## Results

### Genome-Wide Linkage Analysis Revealed a Novel Locus on 4q32

**Figure 1:**
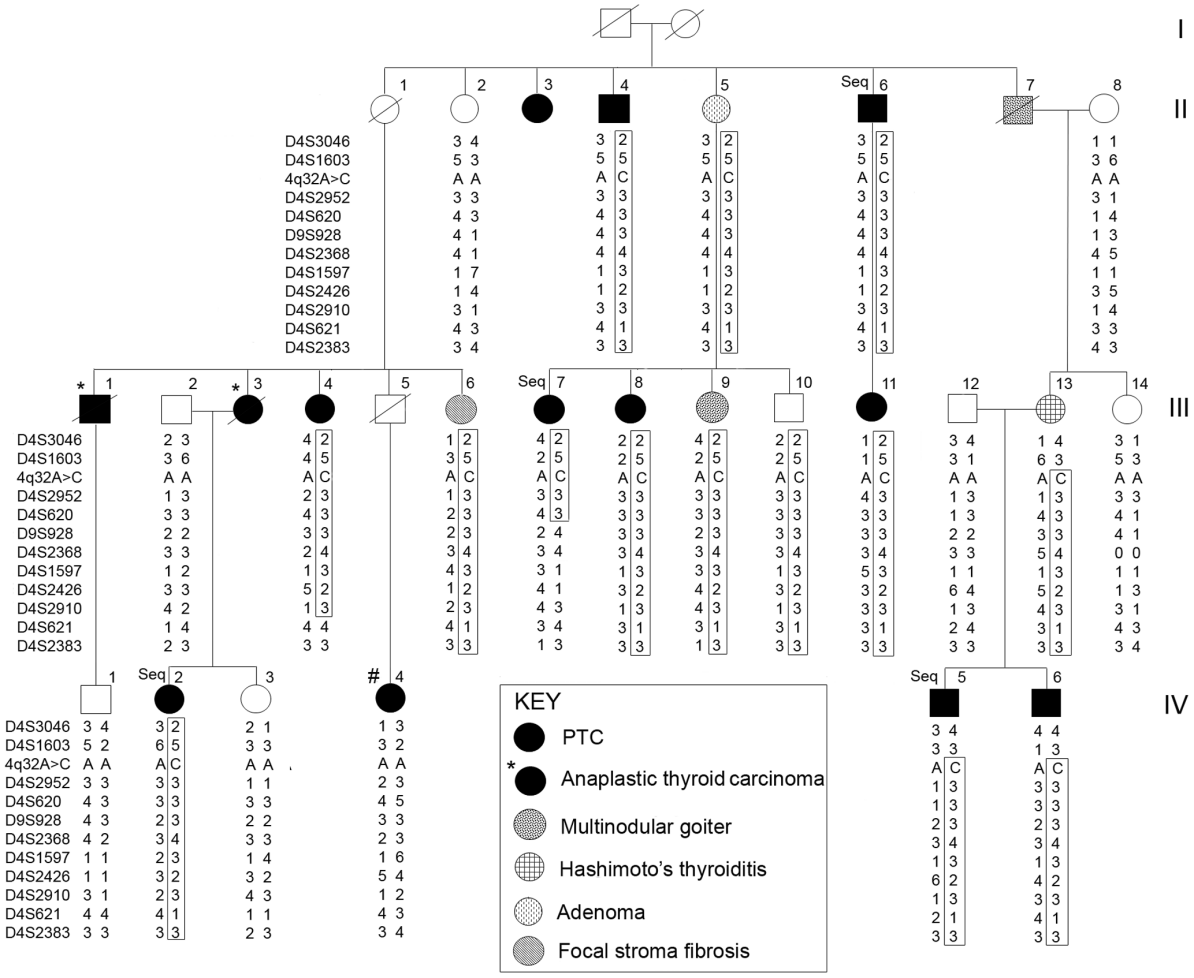
A thyroid cancer susceptibility locus in 4q32. Pedigree showing haplotypes of the family across the 4q32 region. The shared haplotype is boxed. The DNA samples from 4 affected individuals studied by targeted deep sequencing are marked “Seq”. One PTC individual who does not share the haplotype and does not have the 4q32A>C mutation is marked “#”.

The family is an unusually large NMTC kindred with 13 individuals affected with NMTC in at least 3 generations, including 11 cases of PTC (4 follicular variant and 7 conventional) and 2 cases of anaplastic thyroid carcinoma (ATC) (Figure 1). Genome-wide linkage analysis with SNP arrays revealed a locus on chromosome 4q32, with a linkage interval of about 4.6 Mb (from 155.67 cM to 168.2 cM, deCODE map). Multipoint non-parametric linkage analysis yielded a maximum NPL Z-score of 18.5 (see Text S1, Figure S1). To fine map the 4q32 locus, we genotyped 11 microsatellite markers in 22 family members, including 10 individuals with PTC, 4 with benign thyroid disease (two of whom are obligate carriers), and 8 unaffected family members (3 related by marriage) (Figure 1). A shared haplotype segregated with the disease phenotype (thyroid cancer) in all but one affected family member. The haplotype was also found in 4 individuals with benign thyroid disorders; two of whom are obligate mutation carriers. These benign disorders include one case of thyroid adenoma, one case of multinodular goiter and chronic lymphocytic thyroiditis, one case with chronic lymphocytic thyroiditis, and one case with focal stromal fibrosis. The haplotype was not present in 7 out of 8 unaffected individuals (Figure 1).

### DNA Resequencing Identified an A>C Mutation in the 4q32 Locus

Genes within the 4q32 locus include known protein-coding genes (*CPE, KLHL2, ANP32C, SC4MOL*, and *TLL1*) and several uncharacterized transcripts and ESTs (Figure S2). To identify the causal DNA variants/mutations in the locus, we first Sanger resequenced the exons and exon-intron boundaries of all annotated genes. No likely disease-causing DNA changes were found. This prompted us to resequence the entire linkage and flanking regions (∼4.1 Mb) using deep sequencing technology (see Text S1). We enriched the non-repetitive genomic DNA sequences using the *SureSelect* Target Enrichment system (Agilent) and prepared custom paired-end libraries from captured DNA for deep sequencing using the Illumina HiSeq2000 platform. A total of four affected individuals in the family were sequenced as denoted in Figure 1.

To distinguish potentially causal variants/mutations from other variants, we focused only on DNA changes that were shared by the 4 affected individuals and were previously unreported or reported with very low frequency. We found a single relevant-appearing nucleotide change at chr4:165491559 (GRCh37/hg19) in an intergenic region and named it 4q32A>C. We then used Sanger sequencing to examine all the 22 available samples as described above (Figure 1). The 4q32A>C change was present in all affected individuals and segregated with the disease phenotype except for the one family member with PTC who did not show linkage (individual #IV-4). To exclude sampling error or other pre-analytical errors, we Sanger sequenced a second independent blood genomic DNA sample from this patient; the mutation was not found. The 4q32A>C change was also found in 4 individuals with benign thyroid disease and one unaffected individual, but was not present in 7 other unaffected individuals. The 4q32A>C inheritance pattern is consistent with the haplotype analysis using microsatellite markers (Figure 1). For one ATC patient (III-3), we sequenced and genotyped her husband (III-2) and her two daughters, one with PTC (IV-2) and one unaffected (IV-3). Haplotype analysis of these individuals demonstrated that this ATC patient was an obligate carrier of the mutation and the haplotype, since it is present in her affected daughter and her affected siblings but not present in her unrelated spouse (Figure 1).

The 4q32A>C change is not reported in public databases (dbSNP and 1000 Genomes Project). We screened an additional 38 NMTC families (Figure S3), an Ohio cohort of 800 cases/820 controls, and a Polish cohort of 1876 cases/1650 controls. None of these samples showed the 4q32A>C change. We view the 4q32A>C as an ultra-rare variant or a “private” mutation.

### Identification of a Long-Range Enhancer in the Mutation Region

**Figure 2:**
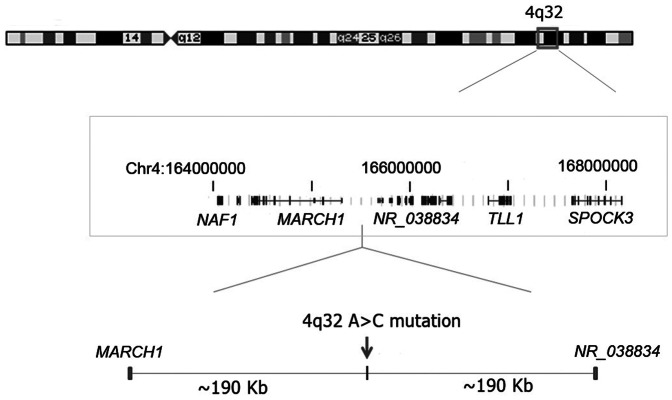
A 4q32A>C mutation in an intergenic region in a large family. Diagram of chromosome 4 showing the area of linkage (boxed) targeted for deep sequencing and the location of the 4q32A>C mutation. Chromosome 4 coordinates and schematic of genes were drawn based on information from UCSC Genome Browser GRCh37/hg19 (not all genes are shown).

The 4q32A>C mutation is located in an intergenic region where the closest known genes are *MARCH1* located ∼190 kb upstream and a non-coding RNA gene (*NR_038834*) located ∼190 kb downstream (Figure 2, Figure S2). We examined comparative genomics data and the ENCODE histone marker data in the UCSC genome browser (Figure S4) and found that the sequence in the 4q32A>C region is highly conserved among mammals, suggesting regulatory element(s) in the region. We then used the enhancer element locator (EEL) computer program to screen for potential evolutionarily conserved enhancer elements [23]. An enhancer element spanning the 4q32A>C mutation site was predicted by the computer analysis (Table S1).

**Figure 3:**
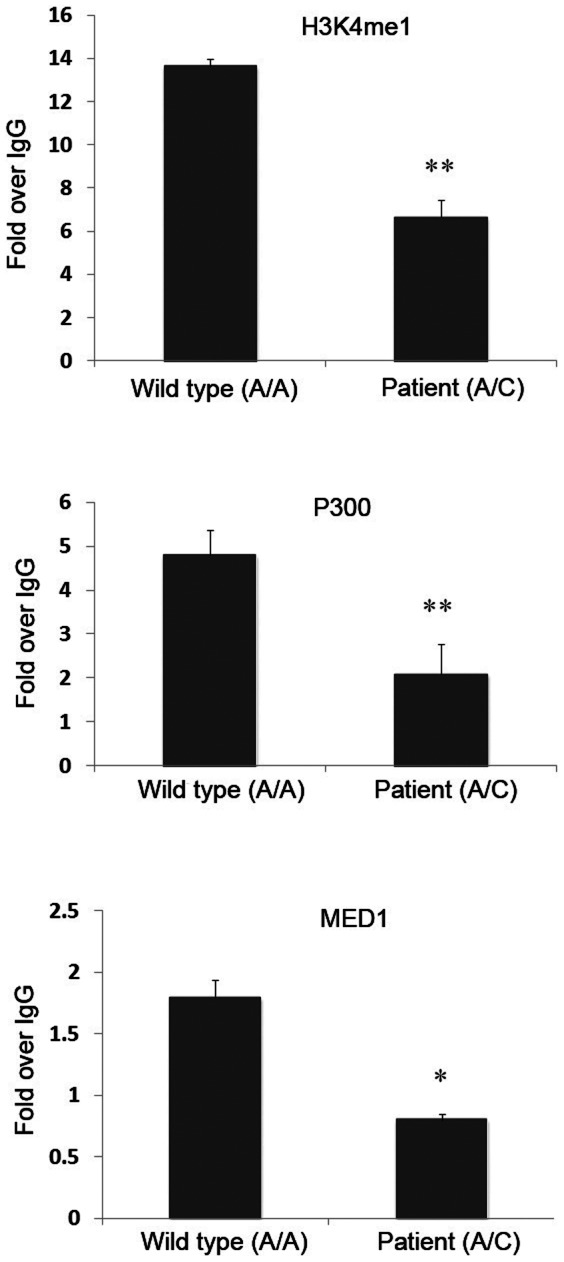
The 4q32A>C mutation is associated with reduced levels of enhancer markers. ChIP assays of lymphoblastoid cell lines established from a control individual (4q32-A/A) and a PTC patient in the family (4q32-A/C). Antibodies against H3K4me1, P300, and MED1 were used in the assays. **: p-value<0.01; *: p-value<0.05. Error bars represent ±SD of at least three independent experiments.

Since the mono-methylation at histone 3 lysine 4 (H3K4me1) and transcriptional co-activators (p300 and MED1) can accurately predict enhancer activity at many loci, we performed site-directed ChIP assays for H3K4me1, P300 and MED1 of lymphoblastoid cell lines established from one thyroid carcinoma patient with the 4q32A>C heterozygous genotype and one control with the wild type A/A genotype. We observed strong enrichment of enhancer mark, e.g.H3K4me1 and robust occupancy of transcriptional co-activators (p300 and MED1) in this region in the control lymphoblastoid cell line (Figure 3). These observations provided independent evidence for the likelihood of strong in vivo enhancer activity from this region. Importantly, these patterns of activity were significantly decreased in the mutant cell line (Figure 3), implying that the enhancer activity is likely damaged or destroyed by the 4q32A>C mutation. To validate these findings, we performed the same assays in a second patient cell line and a control cell line and obtained very similar results, supporting that the enhancer activity is likely damaged or destroyed by the 4q32A>C mutation.

### Functional Impact of the 4q32 Mutation on Enhancer Function

**Figure 4:**
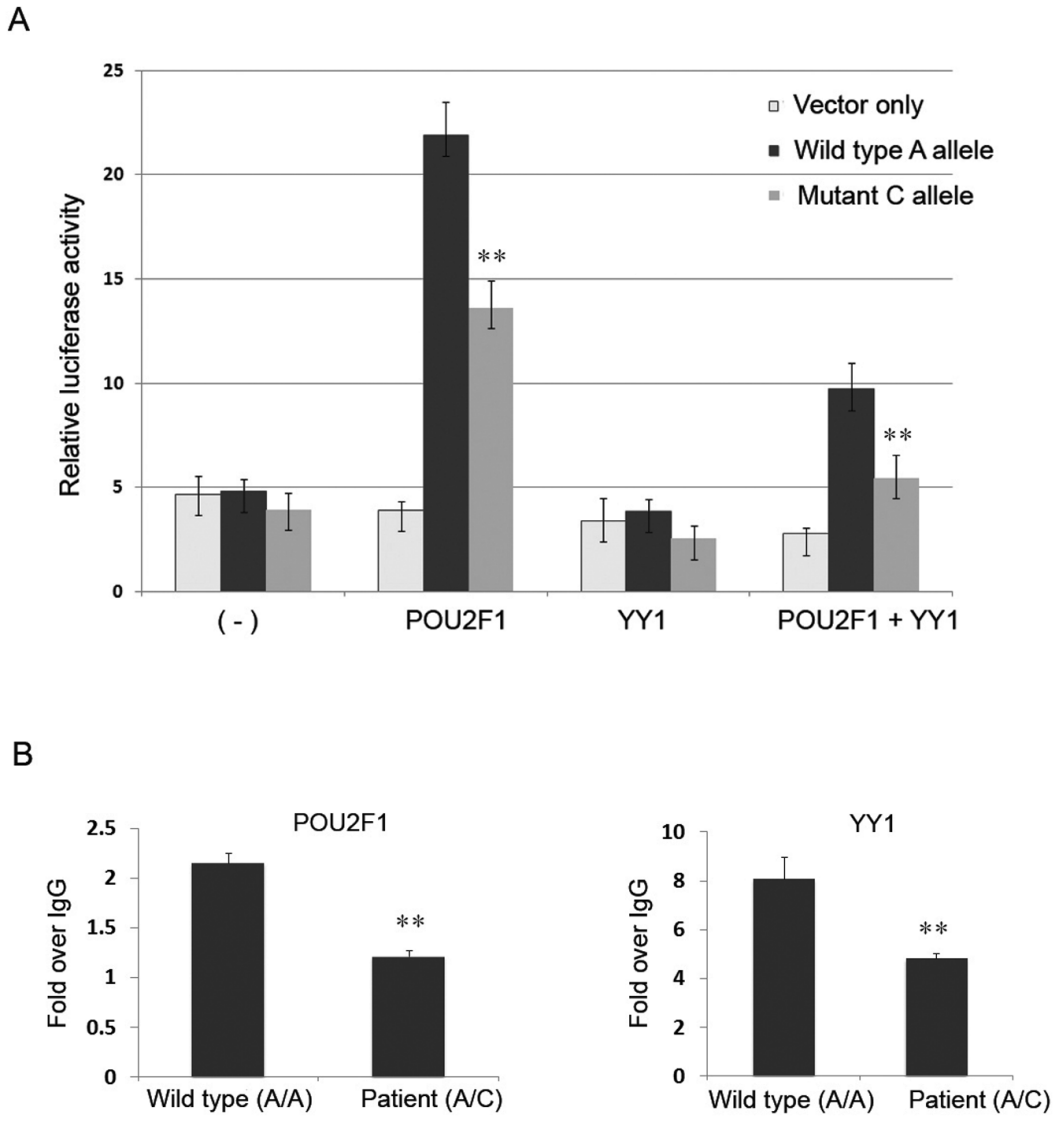
The 4q32A>C mutation leads to reduced enhancer activity and POU2F1 and YY1 binding. (A) Luciferase assays of the enhancer activity using the thyroid cancer cell line BCPAP. **, p-value<0.005. (B) ChIP assay for transcription factors POU2F1 and YY1 using antibodies against POU2F1 and YY1 in lymphoblastoid cell lines. The control and patient cell lines are the same as in Figure 2. **, p-value<0.01. Error bars represent ±SD of at least three independent experiments.

To establish whether the 4q32A>C mutation within the enhancer corresponds to a functional binding site of a known transcription factor, we screened the mutant nucleotide and the adjacent DNA sequence using the TRANSFAC database [24] and identified a composite motif consisting of a potential binding site for transcription factors POU2F1 and YY1 (Figure S5). We hypothesized that the mutant nucleotide might alter transcription factor binding to the 4q32 enhancer and lead to a detectable allele-specific functional effect on transcription. To test this hypothesis, we amplified a 700 bp DNA fragment containing the 4q32A>C mutation using a patient's DNA and cloned the sequence into a luciferase reporter construct driven by a minimal promoter vector. We obtained constructs harboring the wild type A-allele and the mutant C-allele of the 4q32 enhancer, respectively. Enhancer activities were determined by transient transfection and luciferase assays in the thyroid cancer cell line BCPAP. The wild type A-allele had the most pronounced enhancer activity in the presence of a POU2F1 expression construct, while the mutant C-allele showed significantly decreased activity (Figure 4A). We did not observe any effect on the luciferase activity in the presence of YY1 expression construct alone; however, co-transfection of POU2F1 and YY1 significantly inhibited the enhancer activity, compared with that in the presence of the POU2F1 expression construct alone. Moreover, the mutant C-allele showed significant reduction of the luciferase activity in the presence of both POU2F1 and YY1 (Figure 4A). We observed very similar patterns of luciferase reporter activities in the HeLa cell line (Figure S6). These results indicated that the presence of the mutant nucleotide (C-allele) in the enhancer fragment significantly altered the expression of firefly luciferase, suggesting that the chr4 site functions as an allele-specific long-range enhancer and the transcription factors POU2F1 and YY1 are involved in the enhancer activity.

To validate the binding of POU2F1 and YY1 to the enhancer and the disruption of POU2F1 occupancy at the mutated C-allele, we performed ChIP assays and found that the POU2F1 binding was enhanced in the presence of the wild type A/A genotype in a control cell line whereas the binding was significantly reduced in a heterozygous A/C patient cell line (Figure 4B). The binding intensities of YY1 showed a pattern similar to POU2F1, that is, the patient cell line with a heterozygous A/C change showed reduced YY1 binding compared with the control cell line with wild type A/A genotype. We performed the same assays in a second patient cell line and a control cell line and obtained very similar results. Collectively, results of ChIP experiments and luciferase reporter assays are consistent with the hypothesis that the chr4 locus harbors an intergenic long-range enhancer. The 4q32 A>C mutation appears to destroy the intrinsic enhancer activity in thyroid cells and possibly other tissues.

### Transcription at the 4q32 Enhancer and Deregulated Transcription in PTC Tumors

**Figure 5:**
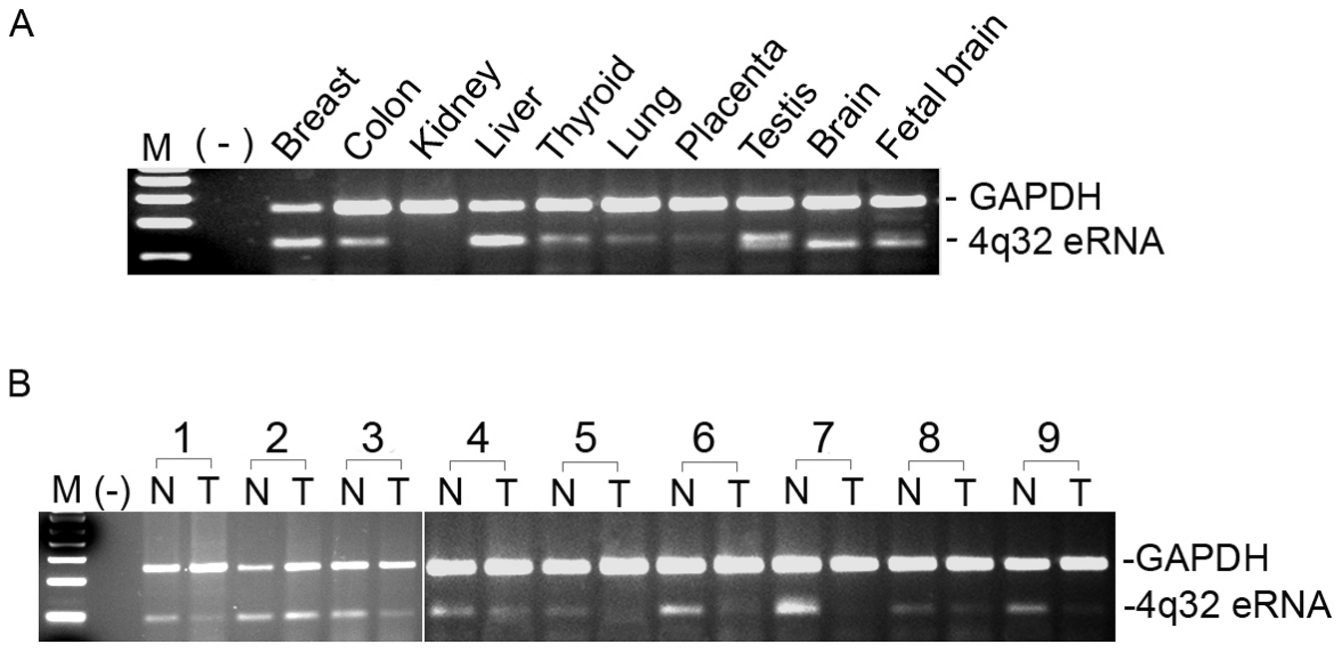
The 4q32 eRNA is expressed in multiple tissues and shows strong downregulation in thyroid tumors. (A) Detection of the 4q32 eRNA in normal human tissues. (B) Semi-quantitative assay of the 4q32 eRNA in paired tumor and unaffected tissue from 9 PTC patients. Total RNA was used for cDNA synthesis; *GAPDH* was used as an internal control. The primers for the amplicons of the eRNA and *GAPDH* are provided in Supplementary Table 2.

To further characterize the 4q32 enhancer, we examined enhancer RNA (eRNA) expression using semi-quantitative RT-PCR. Indeed the 4q32 enhancer produced RNA in normal thyroid tissue (a 134 bp amplicon) (Figure S7). The detection of eRNA in thyroid was not due to the residual genomic DNA that is often present in purified RNA samples, since there was no eRNA detected in the absence of reverse transcriptase. We also detected the 4q32 eRNA in cDNAs derived from human breast, colon, liver, lung, brain, and fetal brain tissue; but not in cDNA derived from kidney (Figure 5A). In addition, we examined the 4q32 eRNA levels in thyroid tumors obtained from 9 sporadic PTC patients and compared them with the matched non-affected thyroid tissue from the same patients. The eRNA level was clearly reduced or undetectable in 7 out of 9 tumor samples using a semi-quantitative RT-PCR assay (Figure 5B).

## Discussion

It is increasingly clear that human populations harbor an abundance of rare variants some of which are deleterious contributing to disease risk [25]. We report here an ultra-rare mutation (4q32A>C) in a large family as the putative causal variant predisposing to non-medullary thyroid cancer. In this particular family, we confirmed this rare mutation in 9 PTC patients and showed that one of the two ATC patients was an obligate carrier of the mutation. ATC is the most aggressive thyroid gland malignancy that is often believed to evolve from PTC [26], [27]. We interpret these findings to suggest that the 4q32A>C mutation is involved in the predisposition to both PTC and ATC in this family. Linkage analysis and co-segregation data suggested relatively high penetrance, and notably, the variant was not found in 38 additional NMTC pedigrees. In addition, it was not found in ∼2500 sporadic PTC cases and ∼2500 controls from two populations, suggesting that this is an ultra-rare, perhaps private mutation. We cannot exclude the existence of other regulatory sequences in the region predisposing to NMTC. Comprehensive enhancer screening in this region is called for.

In addition to those individuals diagnosed with NMTC in the family the 4q32A>C mutation was detected in 4 individuals with benign thyroid disease (two were obligate carriers), which is consistent with previous suggestions that thyroid malignancy and benign thyroid lesions may share the same genetic background under certain circumstances [14], [17]. We also found one unaffected individual carrying the 4q32A>C mutation, and conversely, one individual with PTC who did not carry the mutation. This is consistent with other inherited cancer susceptibility syndromes which are often characterized by age-related, incomplete penetrance and the presence of phenocopies [28], [29]. Thyroid cancer in this family may result from a combination of genetic, environmental, and lifestyle factors, many of which are unknown. Nevertheless, our data suggest that the risk associated with NMTC in this large family is explained by an ultra-rare or “private” A>C mutation in the 4q32 locus. Our work supports the long-established idea that multiple rare variants could be important drivers of common diseases [30]–[35]. This may also contribute to the genetic heterogeneity observed in thyroid cancer predisposition [36].

Increasingly, data are accumulating to suggest that non-coding variants underlie the increased risk of various common diseases [37], [38]. Transcriptional regulatory elements may represent major sites where mutations contribute to human disease [39], [40]. As outlined in the Introduction, high-penetrance genes detectable e.g. by traditional linkage analysis and shared by thyroid cancer families may be uncommon. However our previous linkage and genome-wide association studies have suggested that at least a fraction of the underlying genetic variation associated with NMTC is non-protein coding in nature [17], [22]. In the family presented here, the mutation is located in a long-range enhancer region. Variations in enhancer sequences have been associated with greater risk of several cancers [41]–[48]. For example, the 8q24 locus for colon and prostate cancer appears to harbor enhancers potentially regulating the expression of c-Myc located ∼300 kb away [44], [49]. Similarly, SOX9 has a long-range regulatory element located over 1 Mb from the coding sequence. Genetic variants in the long-range enhancer of SOX9 account for the risk associated with prostate cancer [42]. Common genetic variants at the 11q13.3 renal cancer susceptibility locus influence binding of HIF to an enhancer of cyclin D1 expression [43]. Our finding of a 4q32 enhancer in a thyroid cancer family emphasizes the importance of the role of non-coding regulatory sequences in cancer and the importance of sequencing the entire candidate regions or whole genome when searching for causal variants/mutations in diseases.

Transcriptional regulation is a highly organized process that directs gene expression in response to developmental and signaling events. Disruption of either of these may lead to disease and is certainly known to be associated with malignancy [50], [51]. Key to this control is the recruitment of transcription factors that bind to regulatory sequences, such as enhancer elements. In 4q32, ChIP assays showed that both POU2F1 and YY1 bound to the mutation region and showed decreased binding to the mutant allele compared with the wild type allele. Consistently, we found that the A>C mutation has functional effects in the presence of POU2F1 alone and in the presence of both POU2F1 and YY1 using a luciferase reporter assay. POU2F1 and YY1 are ubiquitously expressed transcription factors that mediate cell-type-specific and temporally restricted gene expression [52]. Both POU2F1 and YY1 are expressed in normal thyroid tissue; they also are involved in the transcription of thyroid specific genes and in thyroid development [53]. We observed decreased binding of POU2F1 and YY1 to the 4q32A>C mutation within the 4q32 locus, which could lead to altered expression of its target gene(s) and to increased susceptibility to NMTC. Many long-range enhancers are tissue-specific and activate their target promoters over long distances, sometimes even on different chromosomes [54]. So far the target genes of most of these enhancers are unknown. We cannot assess how likely, e.g., the neighboring genes, such as *MARCH1* or a noncoding RNA gene (*NR_038834*) might be targets of the eRNA we detected. Systematic assessment of the target gene(s) and their roles in NMTC will be of great future interest. We anticipate that finding target genes relevant to thyroid cancer may be a complex undertaking given the documented multi-organ expression pattern of the eRNA we detected.

In summary, we describe an ultra-rare mutation (4q32 A>C) in a long-range enhancer in a large kindred as a candidate cause of cancer predisposition. Moreover, 4q32 eRNA was detected in normal thyroid tissue and downregulated in thyroid tumor tissue suggesting an involvement of this regulatory element not only in the described family but in sporadic PTC as well. Our finding provides novel insight into the complexity and heterogeneity of the genetic architecture underlying non-medullary thyroid cancer.

## Materials and Methods

### Ethics Statement

The studies were approved by the Institutional Review Board at the Ohio State University, and all subjects gave written informed consent before participation.

### Family Samples and Genomic DNA Extraction

The pedigree of the family is depicted in Figure 1. This large family is of Caucasian, non-Hispanic origin and resides mainly in the mid-western region of the US. The clinical information and family history of all the recruited family members were reviewed. None of the family members have a history of ionizing radiation exposure or any other common environmental exposure that might explain the high rate of thyroid cancer in this family. The histological diagnoses of PTC in 6 PTC patients (II-4, III-4, III-8, III-11, IV-2, and IV-6) were confirmed by re-evaluating the tumor slides by an expert pathologist at the OSU Medical Center. For the 2 cases of ATC in this family (III-1 and III-3), unfortunately, the slides/blocks were not available. The available pathology reports indicated highly invasive and aggressive tumors originating in the thyroid gland in both affected individuals. The tumors had invaded the trachea and esophagus. Both individuals died of their disease within 5–12 months of their diagnosis. An additional 38 families with NMTC in close relatives were also studied. The pedigrees of these smaller kindreds are provided in Figure S3. Family history information, pathology reports confirming the diagnosis of thyroid cancer or thyroid disease, as well as blood and tissue samples were collected from all consenting affected individuals and key unaffected individuals. Genomic DNA was extracted from blood according to standard phenol-chloroform extraction procedures.

### Genotyping and Sanger Sequencing

Genotyping and Sanger sequencing were carried out using standard protocols as described [17]. The PCR primer sequences are provided in Table S2. The PCR assays were performed according to a standard protocol as follows: 2 min at 94°C; followed by 30 cycles of 30 s at 94°C, 30 s at 58°C, and 30 s at 72°C; followed by a final extension of 10 min at 72°C. An ABI 3730 DNA Analyzer was used for either the allele analysis or Sanger sequencing.

### Thyroid Samples and Genomic DNA and RNA Extraction

Fresh snap-frozen thyroid tissue was obtained from patients with sporadic PTC undergoing surgical resection. Control “normal” thyroid tissue was collected from consenting individuals who had surgery because of laryngeal malignancy but no thyroid disease. Clinical data and information on the specimens will be provided upon request. The fresh frozen tissues were selected for study after histological examination. Genomic DNA was extracted by a standard phenol-chloroform procedure. Total RNA from fresh frozen tissue was extracted with TRIzol Reagent (Invitrogen).

### Semi-quantitative RT-PCR

Total RNA was first treated with DNase-1 (Ambion) and then reverse transcribed to cDNA with the SuperScript First Strand Synthesis system (Invitrogen). Candidate genes and an endogenous control gene, glyceraldehyde-3-phosphate dehydrogenase (*GAPDH*) were included in the same PCR reaction for semi-quantitative RT-PCR. All PCR assays were verified to be in the linear range by testing with different cycle numbers.

### Constructs, Cell Culture, Transient Transfections, and Luciferase Reporter Gene Assays

For enhancer-reporter gene assays, a 700 bp intergenic DNA sequence containing the wild type A or mutant C allele in the 4q32 enhancer was PCR amplified and cloned into PGL4.10-E4TATA vector, which contains a 50 bp minimal E4 TATA promoter sequence. These constructs were validated by Sanger sequencing. The BCPAP cell line was incubated in antibiotic-free RPMI1640 medium supplemented with 10% fetal bovine serum at 37°C in humidified air with 5% CO_2_. Co- transfection with empty vector or the enhancer constructs, POU2F1 and/or YY1 expression constructs, and a renilla luciferase reporter plasmid were performed. The empty PGL4.10-E4TATA vector DNA was added if necessary to make sure the same amount of total DNA was used for transfection in all groups. Cells were harvested after 24 h and lysates were used for luciferase assays. At least three independent experiments were performed.

### Quantitative Chromatin Immunoprecipitation (ChIP) Assay

ChIP was performed as previously described [55] with minor modification. Briefly, the Protein-DNA cross-linking was performed by incubating cells with formaldehyde at a final concentration of 1% for 10 min at room temperature. After sonication, chromatin was immune precipitated with specific antibodies at 4°C overnight. The antibodies anti-p300 (sc-585), anti-MED1 (sc8998), anti-YY1 (sc-281) and anti-POU2F1 (OCT1) (sc-232) were from Santa Cruz Biotechnology; Anti-H3K4me1 (ab8895) was from Abcam. The Immune complexes were then eluted from the beads and the cross-links were reversed by incubating at 65°C overnight. The DNA was purified with QIAquick PCR purification kit and used as the template in quantitative PCRs. Primers were designed to yield amplicons ranging in length from 70–110 bp which spanned the mutation region (Table S2).

## Supporting Information

Figure S1 **Linkage plots in the large thyroid cancer family.** (A) Genome-wide linkage analysis with MERLIN; plot of non-parametric linkage (NPL) Z-score in chromosomes 1 to 22. (B) Plot of NPL Z-score in chromosome 4. The linkage peak in chromosome 4 is marked with an arrow.
(TIF)

Figure S2 **The genomic region and genes in 4q32 locus**. Information is obtained from UCSC genome browser (GRCh37/hg19). The position of the 4q32A>C is marked with an arrow.
(TIF)

Figure S3 **Simplified pedigrees of 38 families with NMTC cases**. Members affected with PTC are indicated by filled solid circles or squares. Hatched circles or squares denote those with benign thyroid disease. Open circles: unaffected. For family #26, two members with PTC and melanoma are indicated with two asterisks (**); two members with melanoma only are indicated with one asterisk (*).
(TIF)

Figure S4 **ENCODE histone marker and comparative genomics data from the UCSC genome browser (NCBI36/hg18).** The genomic region encompasses 700 bp; the 4q32A>C mutation is marked with an arrow.
(TIF)

Figure S5 **DNA motifs consisting of potential binding sites for transcription factor YY1 and POU2F1.** (A) Sequencing chromatogram showing the 4q32A>C mutation with DNA sample of a PTC patient; the DNA motif is boxed. (B) The DNA motifs with the wild type A-allele and mutant C-allele in the 4q32 A>C region.
(TIF)

Figure S6 **Luciferase assays showing reduced enhancer activity with the mutant C-allele.** HeLa cells were transiently co-transfected with reporter constructs containing the wild type A-allele or the mutant C-allele with POU2F1 or YY1 constructs. Data shown are the average of at least three experiments. *, p value<0.005. Error bars represent ±SD of at least three independent experiments.
(TIF)

Figure S7 **4q32 eRNA.** (A) The relative position of the eRNA. (B) Detection of 4q32 eRNA in normal thyroid tissues by RT-PCR. M, size marker; (−), no template; RT (−), absence; RT (+) presence of reverse transcriptase. Total RNA was used for cDNA synthesis; *GAPDH* was used as an internal control. The primers for the amplicons of the eRNA and *GAPDH* are provided in Table S2.
(TIF)

Table S1 **Alignment result with Enhancer Element Locator program in the 4q32A>C region.**
(DOCX)

Table S2 **Primers used for sequencing, SNaPshot assay, RT-PCR, and ChIP assay.**
(DOCX)

Text S1 **Supplemental Methods.**
(PDF)
