# Correction: Ultra-Rare Mutation in Long-Range Enhancer Predisposes to Thyroid Carcinoma with High Penetrance

**DOI:** 10.1371/annotation/1cd4e899-2184-497b-b659-de1722b6d402

**Published:** 2013-09-10

**Authors:** Huiling He, Wei Li, Dayong Wu, Rebecca Nagy, Sandya Liyanarachchi, Keiko Akagi, Jaroslaw Jendrzejewski, Hong Jiao, Kevin Hoag, Bernard Wen, Mukund Srinivas, Gavisha Waidyaratne, Rui Wang, Anna Wojcicka, Ilene R. Lattimer, Elzbieta Stachlewska, Malgorzata Czetwertynska, Joanna Dlugosinska, Wojciech Gierlikowski, Rafal Ploski, Marek Krawczyk, Krystian Jazdzewski, Juha Kere, David E. Symer, Victor Jin, Qianben Wang, Albert de la Chapelle

An additional grant from the National Cancer Institute was incorrectly omitted from the Funding Statement. The number of the grant is: R01 CA151979. 

