# Supplementary figures and images for: Ultra-Rare Mutation in Long-Range Enhancer Predisposes to Thyroid Carcinoma with High Penetrance
Source: PLoS One. 2013 May 14;8(5):e61920. doi: 10.1371/journal.pone.0061920 (PMC3653903; doi:10.1371/journal.pone.0061920)

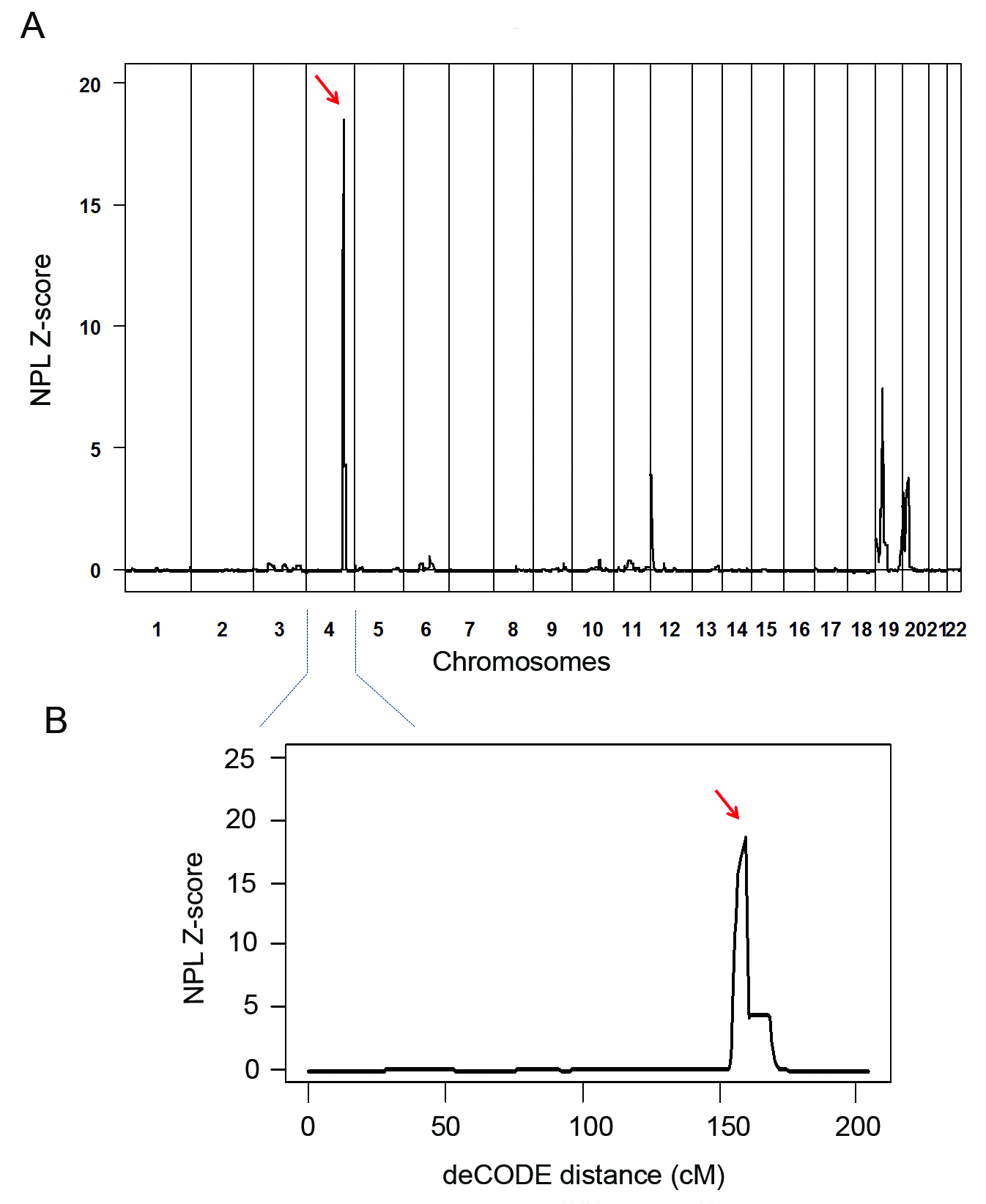

Supplement: Figure S1 — Linkage plots in the large thyroid cancer family. (A) Genome-wide linkage analysis with MERLIN; plot of non-parametric linkage (NPL) Z-score in chromosomes 1 to 22. (B) Plot of NPL Z-score in chromosome 4. The linkage peak in chromosome 4 is marked with an arrow. (TIF) [file pone.0061920.s001.tif]

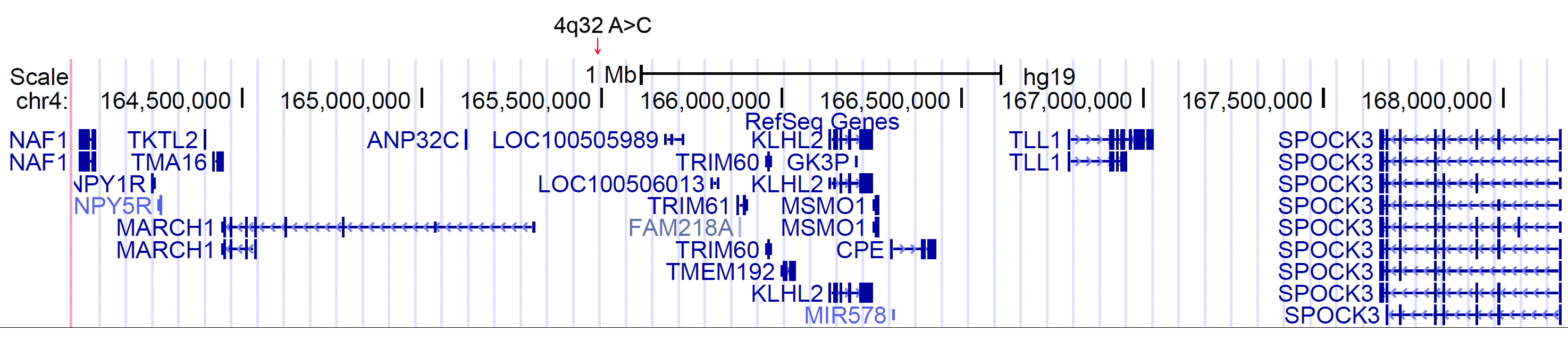

Supplement: Figure S2 — The genomic region and genes in 4q32 locus. Information is obtained from UCSC genome browser (GRCh37/hg19). The position of the 4q32A>C is marked with an arrow. (TIF) [file pone.0061920.s002.tif]

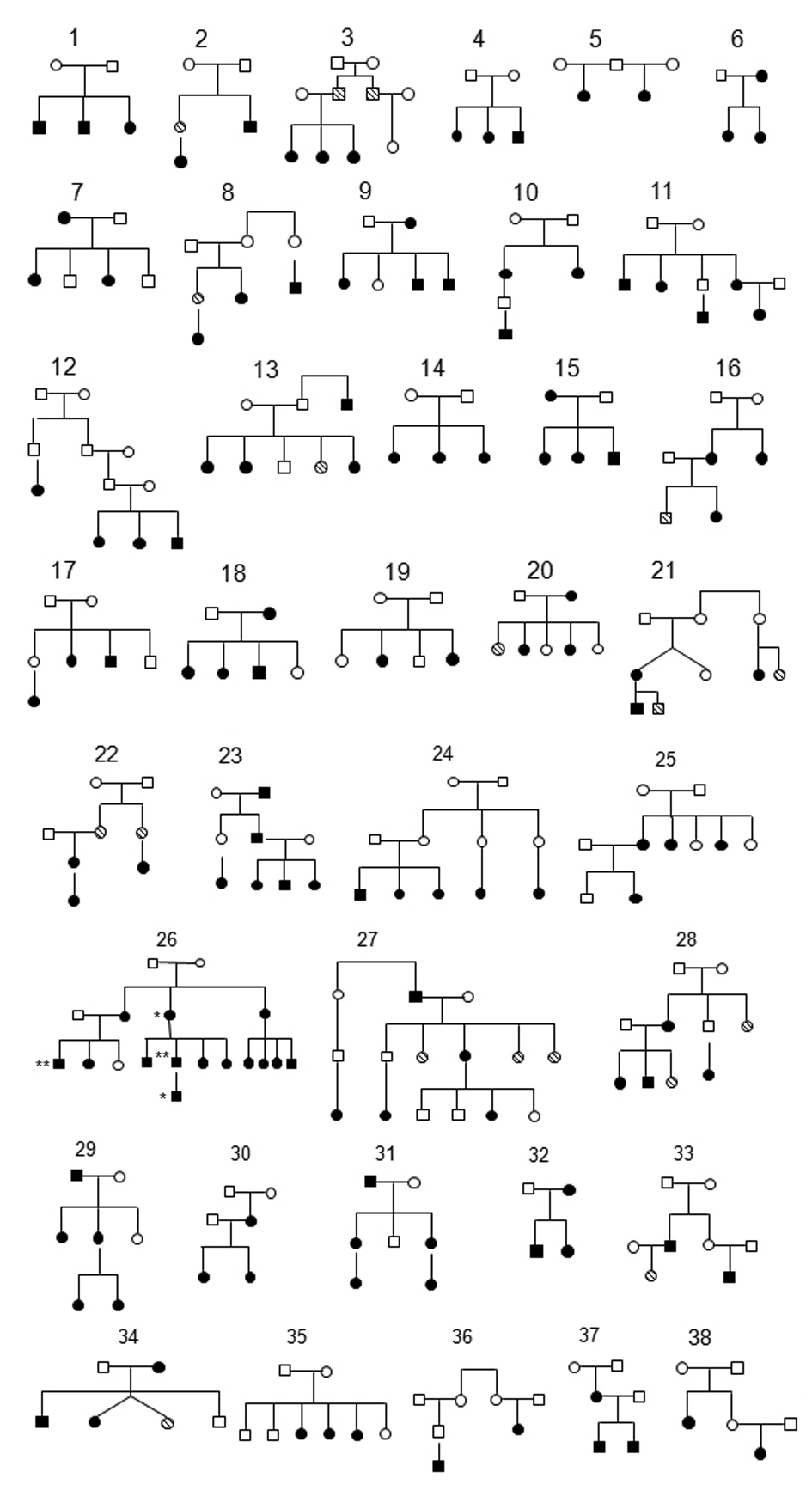

Supplement: Figure S3 — Simplified pedigrees of 38 families with NMTC cases. Members affected with PTC are indicated by filled solid circles or squares. Hatched circles or squares denote those with benign thyroid disease. Open circles: unaffected. For family #26, two members with PTC and melanoma are indicated with two asterisks (**); two members with melanoma only are indicated with one asterisk (*). (TIF) [file pone.0061920.s003.tif]

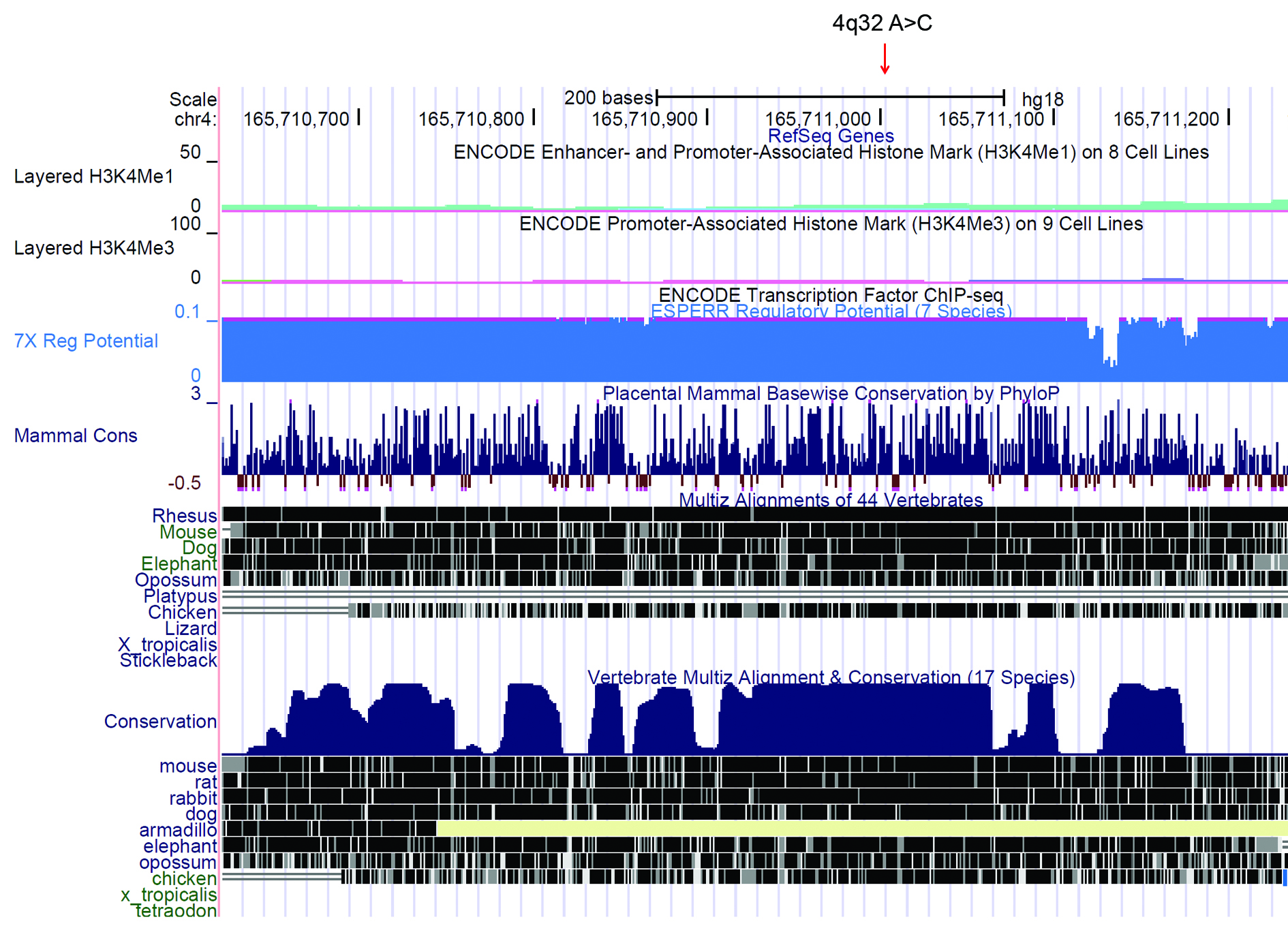

Supplement: Figure S4 — ENCODE histone marker and comparative genomics data from the UCSC genome browser (NCBI36/hg18). The genomic region encompasses 700 bp; the 4q32A>C mutation is marked with an arrow. (TIF) [file pone.0061920.s004.tif]

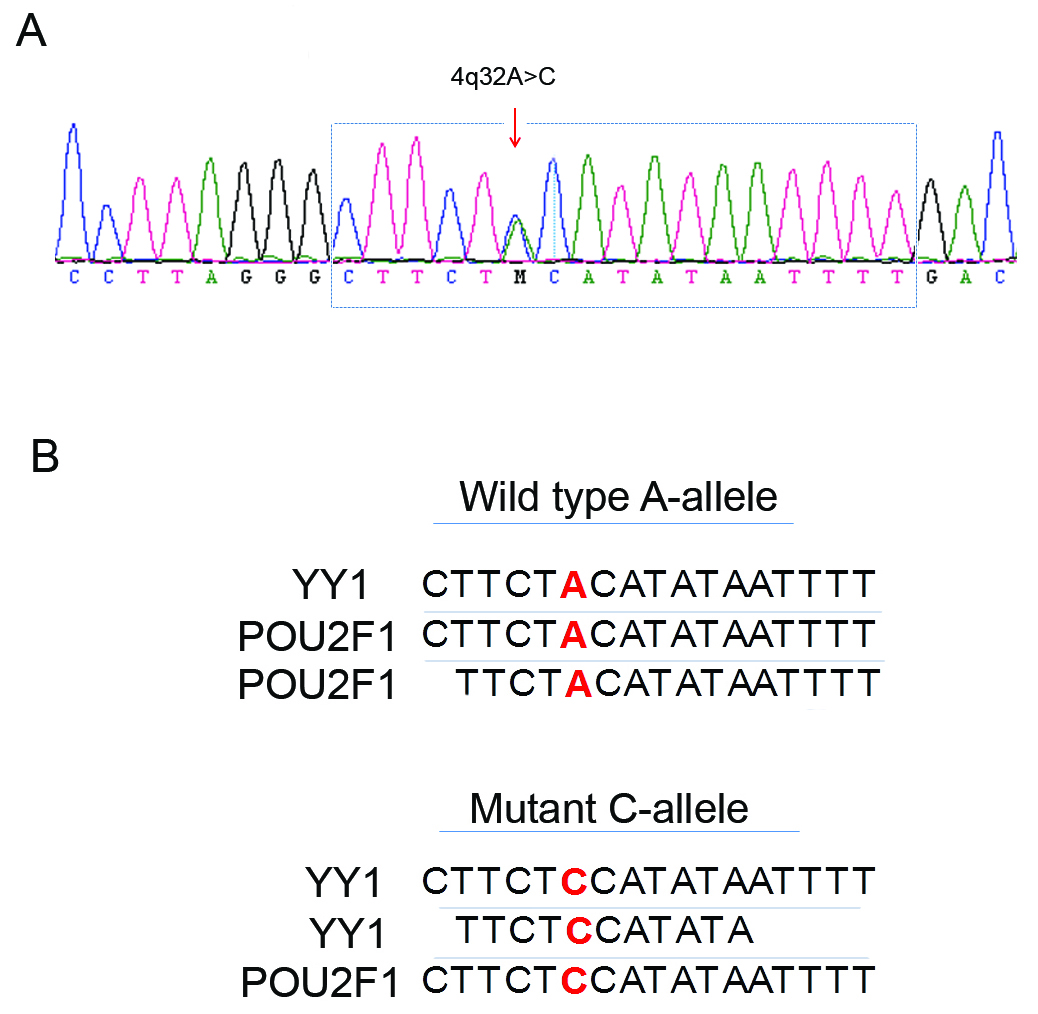

Supplement: Figure S5 — DNA motifs consisting of potential binding sites for transcription factor YY1 and POU2F1. (A) Sequencing chromatogram showing the 4q32A>C mutation with DNA sample of a PTC patient; the DNA motif is boxed. (B) The DNA motifs with the wild type A-allele and mutant C-allele in the 4q32 A>C region. (TIF) [file pone.0061920.s005.tif]

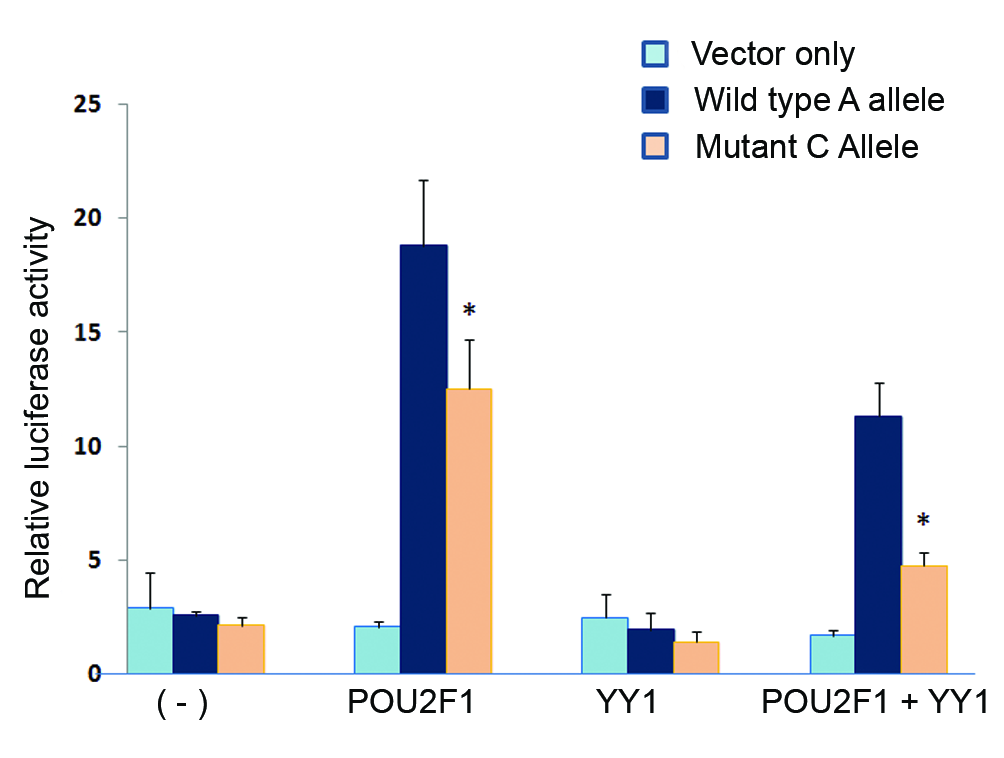

Supplement: Figure S6 — Luciferase assays showing reduced enhancer activity with the mutant C-allele. HeLa cells were transiently co-transfected with reporter constructs containing the wild type A-allele or the mutant C-allele with POU2F1 or YY1 constructs. Data shown are the average of at least three experiments. *, p value<0.005. Error bars represent ±SD of at least three independent experiments. (TIF) [file pone.0061920.s006.tif]

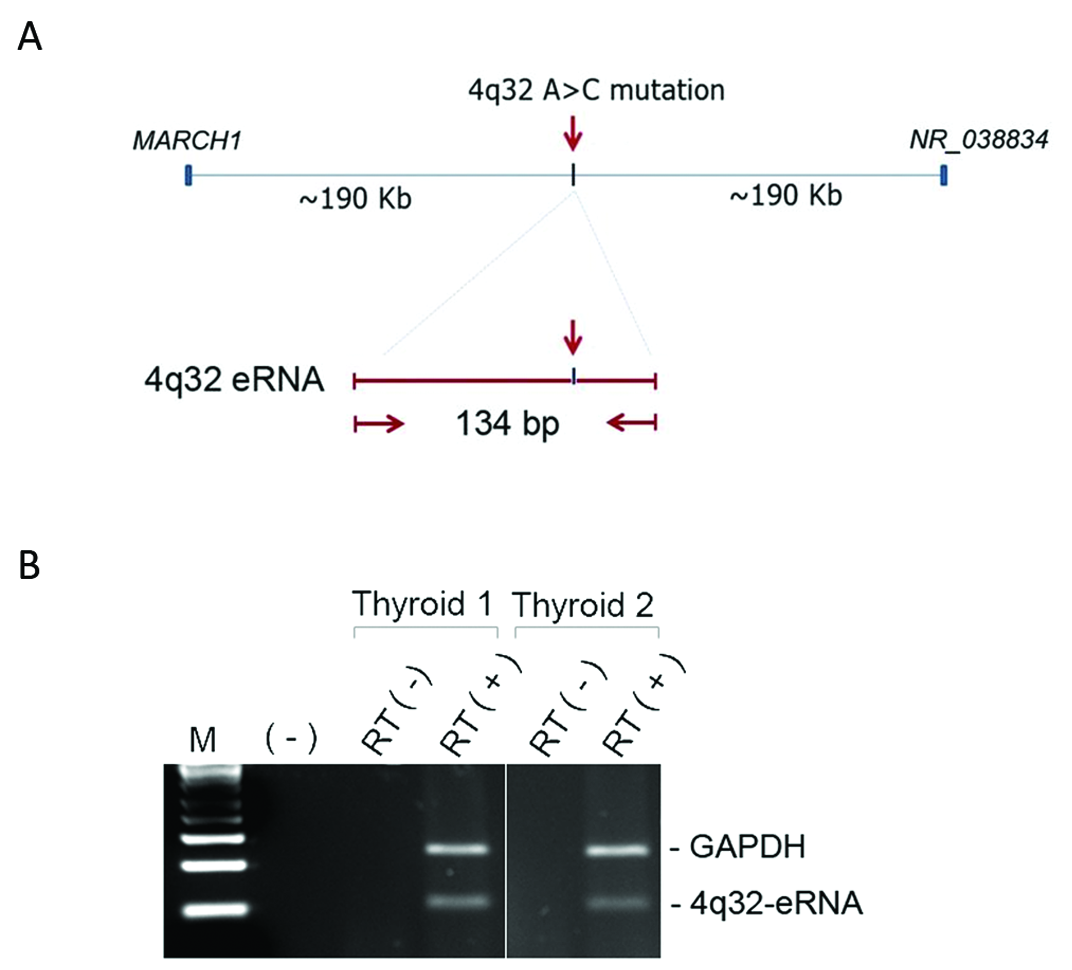

Supplement: Figure S7 — 4q32 eRNA. (A) The relative position of the eRNA. (B) Detection of 4q32 eRNA in normal thyroid tissues by RT-PCR. M, size marker; (−), no template; RT (−), absence; RT (+) presence of reverse transcriptase. Total RNA was used for cDNA synthesis; GAPDH was used as an internal control. The primers for the amplicons of the eRNA and GAPDH are provided in Table S2. (TIF) [file pone.0061920.s007.tif]
