# Supplementary material for: Ultra-Rare Mutation in Long-Range Enhancer Predisposes to Thyroid Carcinoma with High Penetrance
Source: PLoS One. 2013 May 14;8(5):e61920. doi: 10.1371/journal.pone.0061920 (PMC3653903; doi:10.1371/journal.pone.0061920)
Supplement: Text S1 — Supplemental Methods. (PDF) [file pone.0061920.s010.pdf]

Text S1 Supplemental Methods

Non-Medullary Thyroid Carcinoma (NMTC) Families and Genotyping

The large family is of Caucasian origin and resides mainly in mid-western US. An additional 38 families with cases of non-medullary thyroid cancer in close relatives were recruited (Figure S3). Family history information, pathology reports confirming the diagnosis of thyroid cancer or thyroid disease, as well as blood and tissue samples were collected from all consenting affected individuals and key unaffected individuals.

SNP genotyping of genomic DNA from blood using Affymetrix GeneChip Human Mapping Nsp 250K arrays was performed as described [1]. SNP genotype calls were made with Genechip Genotyping Analysis Software (GTYPE) 4.0 (Affymetrix) with default parameters or using the BRLMM program from Affymetrix. The SNP call rate was over 92% with a p value of 0.3. The Mendelian error rate was below 0.2% and errors were removed before analysis.

To screen for the 4q32A>C mutation in populations, BRAF(V600E) mutation in two PTC tumors, and genotyping SNP rs965513 and rs1867277, SNP genotyping was performed using the Sequenom technology (Sequenom) and/or SNaPshot assay (ABI). All assays and data quality assessments were performed according to the manufacturers' instructions.

Genome-Wide Linkage Analysis

Genome-wide nonparametric linkage analysis was performed with MERLIN [1,2]. Calculated allele frequencies based on genotyped individuals were used for linkage scoring. Genetic positions of NPL scores on a chromosome were indicated by using the deCODE map retrieved from Affymetrix NetAffx. The haplotypes were constructed using MERLIN and HaploPainter program.

Targeted Deep Sequencing

In order to capture all genetic variations/mutations in the 4q32 locus, we re- sequenced blood DNA from 4 individuals in the family. Customized sequence capture was conducted by Agilent *SureSelect* enrichment kit to cover the entire linkage peak and flanking regions [3]. Paired-end libraries were sequenced on an Illumina HiSeq2000 platform by the Biomedical Genomics Core of The Research Institute at Nationwide Children's Hospital, Columbus, Ohio. We obtained 30-40 million reads (15-20 million pairs) for each sample, and 100-bp paired-end-reads were aligned to the human genome (hg19) using BWA with default settings. Alignment data were stored in BAM format and visualized using IGV software (Broad Institute). Duplicate pairs were removed by using SAMTools, and only reads with reliable alignment quality

(mapping quality >30) were used to detect variants in the region. Variants were detected using the pileup function of Samtools/BCFtools. ENSEMBL databases were used for gene transcript identification and annotation of amino acid changes.

Bioinformatics Analysis

We used the enhancer element locator (EEL) computer program to screen for potential evolutionarily conserved enhancer elements [4,5] by aligning the human genome sequence to the mouse ortholog (1000 bp with the 4q32A>C mutation site centered). The transcription factor binding site matrices were downloaded from JASPAR [4,6]. The highest scoring element (score 258.42) is a 550 bp long fragment beginning 284 bp upstream of the 4q32A>C mutation site and comprising 14 putative transcription factor binding sites with scores over 200.

Supplemental References

1. He H, Nagy R, Liyanarachchi S, Jiao H, Li W, et al. (2009) A susceptibility locus for papillary thyroid carcinoma on chromosome 8q24. Cancer Res 69: 625-631.
2. Abecasis GR, Cherny SS, Cookson WO, Cardon LR (2002) Merlin--rapid analysis of dense genetic maps using sparse gene flow trees. Nat Genet 30: 97-101.
3. He H, Liyanarachchi S, Akagi K, Nagy R, Li J, et al. (2011) Mutations in U4atac snRNA, a component of the minor spliceosome, in the developmental disorder MOPD I. Science 332: 238-240.
4. Hallikas O, Palin K, Sinjushina N, Rautiainen R, Partanen J, et al. (2006) Genome-wide prediction of mammalian enhancers based on analysis of transcription-factor binding affinity. Cell 124: 47-59.
5. Palin K, Taipale J, Ukkonen E (2006) Locating potential enhancer elements by comparative genomics using the EEL software. Nat Protocols 1: 368-374.
6. Badis G, Berger MF, Philippakis AA, Talukder S, Gehrke AR, et al. (2009) Diversity and complexity in DNA recognition by transcription factors. Science 324: 1720-1723.
