# Supplementary material for: Ultra-Rare Mutation in Long-Range Enhancer Predisposes to Thyroid Carcinoma with High Penetrance
Source: PLoS One. 2013 May 14;8(5):e61920. doi: 10.1371/journal.pone.0061920 (PMC3653903; doi:10.1371/journal.pone.0061920)
Supplement: Supplementary file 3 [file pone.0061920.s008.docx]

Table S1. Alignment result with Enhancer Element Locator program in the 4q32A>C region

Binding site # match	Score	Binding site ID	Binding site position match[a] Mouse <=> Human	Strand
D[107][104]=203.74		/TF/prrx2.pfm	(330,334) <=> (340,344)	+
D[191][190]=240.69		/TF/TOS8.pfm	(428,435) <=> (439,446)	+
D[192][192]=276.22		/TF/FOXO3.pfm	(441,448) <=> (452,459)	-
D[202][203]=300.39		/TF/Che1.pfm	(483,488) <=> (494,499)	-
D[206][204]=338.26		/TF/SIG1.pfm	(492,496) <=> (503,507)	+
D[217][218]=388.93		/TF/TOS8.pfm	(499,506) <=> (510,517)	-
D[224][225]=427.95		/TF/EXD.pfm	(516,523) <=> (527,534)	-
D[229][230]=481.58		/TF/CUP9.pfm	(526,534) <=> (537,545)	-
D[234][236]=517.03		/TF/RUNX1.pfm	(557,567) <=> (568,578)	+
D[239][241]=559.54		/TF/EXD.pfm	(570,577) <=> (581,588)	-
D[247][252]=586.10		/TF/NFIC.pfm	(603,608) <=> (614,619)	-
D[267][265]=596.59		/TF/BTN.pfm	(671,677) <=> (682,688)	+
D[269][271]=635.06		/TF/TEC1.pfm	(686,693) <=> (697,704)	+
D[285][299]=649.76		/TF/MEF2A.pfm	(738,747) <=> (748,757)	+

[a]mm9_1Kb DNA aligned with hg19_1Kb DNA with the 4q32A>C centered.
