# Supplementary material for: Ultra-Rare Mutation in Long-Range Enhancer Predisposes to Thyroid Carcinoma with High Penetrance
Source: PLoS One. 2013 May 14;8(5):e61920. doi: 10.1371/journal.pone.0061920 (PMC3653903; doi:10.1371/journal.pone.0061920)
Supplement: Supplementary file 4 [file pone.0061920.s009.docx]

Table S2. Primers used for sequencing, SNaPshot, RT-PCR and ChIP assay

primers for sequencing & SNaPshot	
4q32_F	cagctggaccactcttctcc
4q32_Rev	tgttacctcccctcctcctt
4q32_SNaPshot-EXT	GAAAGTATCTTTCCTCCTGCCTTAGGGCTTCT
BRAF-For	tgcttgctctgataggaaaatg
BRAF-Rev	CCACAAAATGGATCCAGACA
rs965513-For	tggtgatggtatggtcatgg
rs965513-Rev	cccaggctcaggttatgtct
rs965513-SNaPshot-EXT	gaacagactaatacatcttctttttaatttactc
rs1867277-For	CCGGAGACCAGAGTCCAGT
rs1867277-Rev	ACAGAGGCTCGGGAGTGAC
rs1867277-SNaPshot-EXT	GACCAGAGTCCAGTCCCGGTC
FOXE1 Polyalanin-For	tacagctacatcgcgctcat
FOXE1 Polyalanin-Rev	aggaaccaggccgaagac
primers for eRNA_RT-PCR	
4q32eRNA-Forward	cagctggaccactcttctcc
4q32eRNA-Reverse	ttctttgataaagtaaaggctgtcaa
GAPDH-Forward	aacgtgtcagtggtggacct
GAPDH-Reverse	tgctgtagccaaattcgttg
primers for ChIP assay	
4q32A>C-forward	CCTCCTGCCTTAGGGCTTCT
4q32A>C-Reverse	GATATTCAACCACAGTAAGGAAGGCT
